# Inside the Minds of Professional and Elite Athletes: A Qualitative Exploration of Perspectives on Cannabinoid Use in Contact Sports

**DOI:** 10.1177/19417381261460194

**Published:** 2026-07-07

**Authors:** Elizabeth S. Thompson, Jane Alcorn, Robert B. Laprairie, Noelle Rohatinsky, Payam Dehghani, J. Patrick Neary

**Affiliations:** † University of Saskatchewan, Saskatoon, Canada; ‡ University of Regina, Regina, Canada

**Keywords:** athlete education, cannabinoids, cannabis, elite athletes, qualitative research, stigma

## Abstract

**Background:**

As cannabis legalization expands and professional sports organizations reassess cannabinoid policies, elite contact sport athletes are increasingly exposed to cannabis use for therapeutic, recovery, and recreational purposes. Despite these shifts, limited evidence-based education and persistent stigma may hinder informed decision-making and athlete-clinician communication.

**Hypothesis:**

Professional and elite-level contact sport athletes exhibit inconsistent knowledge and perspectives regarding cannabis use, shaped by stigma, inadequate education, and regulatory ambiguity.

**Study Design:**

Qualitative descriptive study.

**Level of Evidence:**

Level 4.

**Methods:**

A total of 10 semi-structured interviews were conducted with current or former elite contact sport athletes from professional and collegiate leagues, including the National Hockey League, National Football League, National Basketball Association, American Hockey League, National Collegiate Athletic Association, and USports. Interviews explored athletes’ knowledge, attitudes, and experiences related to cannabis and cannabinoid products in sport. Transcripts were analyzed using Braun and Clarke’s 6-step thematic analysis framework to identify recurring patterns and themes.

**Results:**

A total of 5 primary themes emerged: (1) athletes use cannabis; (2) insufficient education and lack of credible information sources; (3) persistent stigma and fear of judgment; (4) limited trust and communication with medical staff; and (5) inconsistency in laws, policies, and organizational responses. Athletes reported cannabis use for pain management, sleep, recovery, and mental health, typically with deliberate control over dosage, timing, and administration methods. Across interviews, participants emphasized the need for accessible, evidence-based education for both athletes and medical personnel.

**Conclusion:**

Elite contact sport athletes use cannabis intentionally for recreational, therapeutic, and recovery purposes but face systemic barriers related to misinformation, stigma, and inconsistent policy guidance. Enhancing athlete and clinician education and standardizing cannabis-related regulations across leagues may support safer, evidence-informed practices.

**Clinical Relevance:**

This study identifies critical educational and policy gaps regarding cannabis in elite sport and underscores the need for multidisciplinary collaboration to guide responsible cannabinoid use among athletes.

The adequacy and availability of quality education regarding cannabis use for elite contact sport athletes is currently unknown. As cannabis legality expands, this potential knowledge gap impairs athletes’ ability to make informed decisions about cannabis for health and performance. Lack of knowledge increases exposure to unregulated products,^7^ and may cause athletes to overlook potential therapeutic benefits.^42^ Poor communication with medical teams heightens misuse risk.^21^

Healthcare curricula rarely include cannabis education, leaving providers with little understanding of its mechanisms or therapeutic potential. Research found only 9% of schools offered cannabis therapeutics, with nearly 90% of medical students feeling ill-equipped to recommend cannabis therapies.^20^ This gap leaves athletes without appropriate medical oversight, while profit-driven commercial markets fill the information void instead of knowledgeable clinicians.^30^

Cannabis is often accessed without healthcare provider involvement. In Canada, the 2023 Medical Cannabis Access Survey noted that 68% of those previously authorized medically now purchase from the recreational market without oversight.^8^ With sports leagues removing cannabis from the banned substance list, athletes increasingly rely on personal experience or peer advice to guide them, with minimal formal education ever provided.^15^ This study examines professional and elite-level contact sport athletes’ knowledge, perspectives, and experiences regarding cannabis in sports. Results can guide league resource allocation, support further research, and improve access to information on cannabinoid rules and safety for athletes and medical staff. Cannabis legislation changes are reflected in evolving league policies. While the National Basketball Association (NBA),^28^ National Hockey League (NHL),^38^ and Major League Baseball (MLB) have reduced prohibitions, the National Football League (NFL) and the World Anti-Doping Agency (WADA) retain restrictions; however, the NFL recently funded research on cannabinoid therapy for pain, concussion, and opioid use.^37^ This qualitative study provides athletes knowledge and perspectives on cannabinoid therapy in elite sport.

## Objectives of the Study

This study aimed to characterize the experiences and perspectives of elite contact sport athletes regarding cannabinoid use, to examine the prevalence of use and the factors motivating it, to identify the principal sources of cannabinoid-related information and athletes’ interest in cannabis education, to explore perceived stigma and barriers to accessing reliable information, and to evaluate communication between elite athletes and their medical support staff. We hypothesized that professional and elite-level contact sport athletes would have variable knowledge and perspectives regarding cannabis use, potentially influenced by stigma, limited education, and regulatory ambiguity.

## Methods

Semi-structured interviews were conducted with professional or college-level contact sport athletes under guaranteed confidentiality. This work was approved by the University of Saskatchewan Behavioral Research Ethics Board (Application ID 3889).

### Participants

We recruited 10 contact sport athletes (aged 19 to 49 years) who competed at the professional or collegiate level with experience in the NHL, NFL, NBA, American Hockey League (AHL), National Collegiate Athletic Association (NCAA), or USports. Recruitment occurred via social media by posting advertisements to participate in a survey and interview for research, and university settings in the form of posters and student newsletters.

### Data Collection

A semi-structured interview guide (Supplement 1, available in the online version of this article) provided flexibility while addressing key topics including personal experiences with cannabis, cannabis education, perceived safety of use in sport, the role of legalization, and communication barriers with medical staff. Interviews lasted 30 to 45 minutes each.

### Procedure

The same researcher conducted all interviews via Zoom video conferencing (Zoom Video Communications Inc (2024) Zoom), enabling participation across locations and leagues. With informed consent and assurances of confidentiality, interviews were audio-recorded. To ensure confidentiality, transcripts were deidentified. Participants were encouraged to speak freely and expand on open-ended questions they found most relevant. Recordings were transcribed verbatim using Zoom transcription software and then checked manually for accuracy. Transcripts were imported into NVivo 14 (Lumivero (2023) NVivo (Version 14)) for systematic organization and analysis.

### Data Analysis

Utilizing Braun and Clarke’s 6 steps of thematic analysis, themes in the data were identified and developed.^14^ These steps include:

1. Familiarization: reading and re-reading the transcripts, noting initial ideas.
2. Generating initial codes: systematically coding interesting features across the dataset using an inductive approach.
3. Searching for themes: collating codes from the codebook into potential themes
4. Reviewing themes: ensuring themes work in relation to the coded data and entire dataset.
5. Defining and naming themes: refine each theme and generate clear definitions and names for each theme.
6. Producing the report: selecting compelling extract examples, conducting a final analysis and relating the findings back to the research question and literature to produce a scholarly report of the analysis.

### Researcher Reflexivity Statement

As the primary researcher with 25 years’ experience in professional and elite sports and academic expertise in cannabinoids, I recognize my background influences my role in this study. While this knowledge helps contextualize athlete experiences, it was intentionally bracketed to keep participant perspectives central. I hold a neutral stance on cannabis, having observed both positive and negative effects, and approached this work to understand athlete perspectives and improve education amid evolving cannabis laws and sport policies. Reflexive thematic analysis was used to enhance transparency, identify potential biases, and maintain rigor throughout the research process.

## Results

The interviewed athletes were aged 19 years to 48 years, with an even distribution of current, recently retired, and long-retired participants. There were 8 male and 2 female athletes included in the sample. Athletes had a mean playing career of 5.1 years (SD, 2.9) and were based in Canada or the United States. Their experience with cannabinoid products ranged from extensive to none, but all had been exposed to cannabinoid products within their team or league context.

Five primary themes were identified from the interviews, with subthemes within most primary themes (Table 1).

**Table 1.** Primary themes and subthemes

Primary themes	Subthemes
Athletes use cannabis	• Why athletes use cannabis • Cannabis use patterns • Cannabis in athletic culture
Education and knowledge	• How athletes learn about cannabis • Reasons athletes want cannabis education • Medical staff need cannabis education
Fear of judgement and punishment	• Negative stigma and punishment • Complexities of trusting medical staff
Hiding in plain sight	
Law and order	• The influence of rule and law • Organizational issues

### Athletes Use Cannabis

The theme “Athletes Use Cannabis” confirms that athletes do not avoid this substance, and explores why and how elite athletes use cannabis and how this use is embedded in sport culture. Athletes describe motivations including pain management, mental health support, relaxation, recovery, sleep, perceived neuroprotective benefits and preferring cannabis to alcohol in social situations. They describe deliberate decisions around timing, frequency, products, and doses, and reflect on stigma, judgment, internal conflict, and positive social contexts. Together, these accounts highlight a complex relationship between athletes and cannabis across 3 subthemes: “Why athletes use cannabis,” “Cannabis use patterns,” and “Cannabis in athletic culture.”

#### Why Athletes Use Cannabis

##### Pain Management

Pain relief is a central motivation for cannabis use. One athlete explained, “It [cannabis] makes you focus on other things, or just not being focused on how bad my neck would hurt” (EA1). Another noted, “It puts your body at ease, and it’s far better than taking any sort of pain killer in my experience, from a prolonged long-term perspective for sure” (EA8). CBD tinctures and oils were popular for musculoskeletal pain.

##### Mental Health

Athletes described cannabis use for anxiety, depression, and stress. One reported shifting from alcohol, “I stopped drinking alcohol because of the adverse effects. My freshman year I consumed a lot of alcohol, and I found it was really bad for both my mental and physical health. I think I wanted to explore an alternative. I wanted to try both the therapeutic and recreational [use of cannabis]. It was just kind of experimental” (EA10). Another emphasized a needed “pause button”: “We’re high strung, right? And sometimes we just needed that pause button that just slows us down. . .that’s what weed did for us at that time” (EA9).

##### Recovery, Relaxation, and Sleep

Cannabis was seen to promote physical and emotional relaxation, aid recovery from long seasons and heavy training, and improve sleep for performance. One athlete observed, “There have been people that use [cannabis] both in season and out of season for recovery purposes. . .As a professional athlete, you always wanna try to recover as fast as possible” (EA2). Another described mind-body effects: “It has always been something that I found meditative and grounding, the mental spiritual components of it, and then, physically it puts your body at ease” (EA8). Sleep benefits were highlighted: “The positive effects I felt [from cannabinoids] with sleep and rest is definitely good” (EA7). Another contrasted cannabis with prescribed analgesics: “I sleep better using cannabis at night than I did taking T3s [Tylenol with codeine]. . . a better effect on my health when it comes to sleep dealing with inflammation and arthritis” (EA6).

##### Neuroprotection, Cognition and Social Use

Some athletes perceived cannabis as neuroprotective, especially after concussions. One shared, “I totally used cannabis throughout having those concussions and it helped me a lot with the pain and the recovery” (EA5). Athletes also used cannabinoids to aid concentration: “It helped me focus a lot. Sometimes I would use it in the spring months when I had exams, and it would help me really dial in on a video or on a subject” (EA10). Many used cannabis to enhance enjoyment and social experiences, sometimes instead of alcohol. One recalled, “When I used it [cannabis] recreationally, I just felt happier and more giddy and definitely just way more inclined to laugh. . .I just felt a lot lighter. My mood felt so uplifted” (EA10). Another described team use: “. . . a bunch of guys on the team were cannabis users, and generally would smoke after practice, or if we had some team parties. . .cannabis use was acceptable totally there” (EA5). Several viewed cannabis as a safer or preferable alternative to alcohol: “The talks have definitely begun of switching. I know some people they prefer not to drink at all, and that [cannabis] is something they do recreationally instead” (EA2).

#### Cannabis Use Patterns

##### Timing and Dosing

Athletes consider how cannabis fits around training, with some abstaining during season. One stated, “I have not used any [cannabis] at all” (EA2). Others used after exertion: “After the gym when I’d be sore, my friends and I would smoke” (EA1). Athletes describe deliberate choices about cannabinoid content, dose, and delivery route to achieve desired effects and avoid overuse. One emphasized, “I’m quick to point out why I use it [cannabinoids], and that I’m not using it for any psychotropic effects” (EA7). Another highlighted balancing cannabinoids: “I need a better balance [of cannabinoids] to really function on the highest level. . .I always like to mix that [CBD (cannabidiol)] with my THC [tetrahydrocannabinol]” (EA5). Many prefer nonsmoked routes such as edibles, tinctures, gummies, and topicals, often due to respiratory concerns. One explained, “Edibles and tinctures were something I just experimented with, and I found really positive benefits of both of them in low doses” (EA10); however, others favor smoking for the rapid onset during recovery or training‑related use.

##### Frequency

Frequency ranges from occasional to daily, with some athletes adjusting intake in‑season to support performance. One stated, “I don’t consume a lot. I consume a very low dose, so I think that that’s important to consider” (EA10). Another said, “I started using it [cannabis] more. It started to be a more routine, everyday type thing. . .” (EA9). Some described self‑correction when use felt counterproductive, recalling that regular use could pull them away from extra work at practice and that they adjusted patterns to stay aligned with performance goals.

##### Peer Influence and Personal Choice

Peers can introduce or normalize cannabis, but athletes also stress personal responsibility and bodily autonomy. One admitted, “The time [I used cannabis] in college honestly was just because they [teammates] were doing it” (EA3). Others highlighted controlled, private use: “I was never out of control of my body [when using cannabis]. . .I would take it [cannabinoids] in a very calm and safe setting just within my apartment” (EA10). Personal history also shaped patterns, with some using cannabis from adolescence and continuing into their careers.

#### Cannabis in Athletic Culture

##### Judgment, Self-Judgement and Image

Athletes often anticipate or experience judgment from teammates, organizations, and the public, especially in shared spaces such as hotel rooms and locker rooms. One athlete described worrying about a roommate’s reaction: “You’re questioning ‘Well, did he see me take it [cannabis]?. . .Did so and so rat on me to the coaches?’” (EA2). Another felt teams would view cannabis use negatively and that even asking about it could be risky. These pressures can become internalized stigma. One athlete reflected, “I judged myself as well. I definitely had a lot of guilt surrounding my use. . .that stigma caused me to use more [cannabis] and rely on it more in secret” (EA5). They spoke about feeling mischievous or “bad” when using cannabis and worrying that being labeled a “cannabis user” would overshadow their broader identity and achievements. “It’s the image society gives cannabis users as a law breaker and a rebel, and someone who’s a bad person in general. I identify with cannabis and if cannabis wasn’t so connected to that [negative image] and the culture, I wouldn’t have had any real reason to identify with the bad aspects of being a rebel right? . . . if cannabis was just acceptable as a medicine right off the bat, then I would have been cool seeing myself that way” (EA5).

##### Team Control and Autonomy

Athletes described organizational control, including fear of sanctions and emphasis on team image. One recalled, “There was the idea that if you get caught with cannabis then you’re charged. . .They [the team] are more worried about the PR [public relations] nightmare and how it looks for the team and the community” (EA6). Another described experiences of “total control” by coaches and teams, with strict consequences that discouraged use for most players. In contrast, several athletes emphasized cannabis as a legitimate option alongside conventional treatments. One athlete argued that athletes should be able to decide with their families which therapies to pursue and questioned reliance on other pharmaceuticals that can be difficult to discontinue (EA3).

##### Social Connection and Shifting Norms

Despite stigma, many athletes report positive, often discreet social environments around cannabis use. One noted, “Yeah, people knew [I used cannabis]. And the majority of my team used it also” (EA1). Another contrasted cannabis gatherings with alcohol‑focused nights out, describing smaller, calmer settings where teammates relaxed, listened to music, and “tried not to be seen.” Athletes also perceive a generational shift, with younger athletes more accepting of cannabis and discussing it more openly with peers, coaches, and trainers. One commented, “I do see in the future there being more of a shift towards cannabis” (EA10). Others described conversations about “switching” from alcohol to cannabis in social settings (EA2), partly because cannabis‑centered environments feel calmer and less likely to lead to conflict or regrettable decisions. They also described the contrast in next day effects between cannabis and alcohol, “ I feel way worse from a physical standpoint the following day when I consume alcohol [compared with when cannabis is used], my recovery time feels much longer and I feel weaker. I don’t feel great at all. . .I feel I am way less in control of my speech, of my thoughts, of my actions [with alcohol].” (EA10) Some athletes reported negative experiences with cannabis, including self‑blame for poor performance or disciplinary consequences, but felt punitive responses such as suspensions could worsen mental health and increase use, and that education or counseling would be more constructive.

### Education and Knowledge

The theme “Education and Knowledge” describes how athletes acquire and use cannabis information. Three subthemes were identified: “How athletes learn about cannabis,” “Reasons athletes want cannabis education,” and “Medical staff need cannabis education.”

#### How Athletes Learn About Cannabis

Athletes learn through varied sources including peers, the internet, and personal experimentation, with limited formal guidance. One athlete explained, “None of my coaches or trainers, or doctors had any resources or knowledge regarding the subject” (EA10). Another pursued scientific literature: “I’d find PubMed, I’d find academic papers on it” (EA8).

Peer influence played a central role. Athletes turned to teammates, friends, and family, describing their sources as “users, peers that have experience, whether it be good or bad or different” (EA4) and seeking advice from people who “understood the industry more” than they did (EA6). These conversations, including with high‑profile users, prompted questions such as, “What are the benefits to this [cannabis]? And why are these other people that have had amazing careers. . . using something like this?” (EA8). At the same time, athletes recognized the risks of relying on peers in the absence of clear guidelines: “There is [sic] no real guidelines on how much [cannabis] you should be taking, when you should be taking it, and how often. . . if you don’t have the knowledge from someone teaching you, it could leave people kinda stranded. . . and if you don’t have guidelines on what it looks like when you do need help for that [cannabis use disorder]. . . that can lead to big trouble” (EA2). 

Self‑experimentation was common. Athletes tried different products and dosages, adjusting to personal responses and competition schedules: “We just went and got it [cannabis] from whoever we knew locally. . . I just took it [cannabis] little by little and took a little bit more when I didn’t have a game” (EA9). Others became familiar with commercial products “by using the products” and “just seeing what works and what doesn’t work” (EA2, EA5). Healthcare practitioners were rarely consulted, and team presentations mainly framed cannabis as a negative and harmful substance which “honestly just felt just like persuasion tactics” (EA4, EA6).

#### Reasons Athletes Want Cannabis Education

Athletes want education tailored to their physical and mental demands, particularly around performance, recovery, and health, noting they were “never actually told what it [cannabis] can do to our health” (EA10). They also asked for structured sessions like other preseason presentations, so staff and players could learn about cannabinoid options openly without feeling they had to “go through the back door and sneak around people” (EA2).

If cannabis rules changed to allow use, athletes suggested prioritizing education, hoping medical staff and management would initiate conversations and provide “some sort of education module about how to consume it responsibly,” emphasizing that responsible use would be crucial (EA10). Curiosity about cannabis’ effects and detection further motivated learning, as athletes were unsure whether it would help or hinder performance or how long it would stay in their system, especially relative to training demands and testing (EA2, EA9).

Several athletes described seeking information to manage health, avoid abuse, and understand past use, including one who believed earlier education about chronic THC use, concussions, and memory could have supported academic performance and mental health (EA5). Others reported discovering CBD only later and felt earlier guidance could have supported faster recovery and improved wellbeing during their competitive years (EA3).

Public visibility added pressure to be well informed so they could respond to scrutiny and parental questions, with one predicting that if a major league allowed cannabis “so many people [would be] freaking out about it” and called for “education across the board for everyone” (EA3). Harm reduction was a key motivator, as athletes wanted guidance on safe, effective use and “harm prevention,” with some preferring to “trust somebody else. . . to learn that stuff and to advise me” rather than complete a formal course themselves (EA7, EA9).

Participants reported that the limited education they received from teams or governing bodies was largely 1‑sided, focused on negative effects and rarely clarified the rationale for cannabis rules, leading them to question why alcohol was permitted while cannabis remained prohibited and noted that annual “safe sport” trainings seldom addressed cannabis specifically (EA1, EA3, EA4, EA10). Athletes argued that punishment for cannabis use should incorporate counseling and education, rather than rely solely on suspension, recalling cases where players were simply removed from competition instead of being “educated about cutting back.” Athletes suggested “something that they go to, just like counseling to get educated,” would feel “so super cool” and change how they viewed both discipline and cannabis use (EA5, EA9).

#### Medical Staff Need Cannabis Education

Athletes reported that medical staff lacked cannabis knowledge and experience, and showed little interest in learning, viewing it as “more of an unknown” that sport culture avoided because of “confusion” (EA2). “Older” trainers and clinicians were seen as particularly resistant to change, shaped by “war on drugs” messaging and long‑standing reliance on traditional medications, with some “grow[ing] up with weed being a bad thing” and a “gateway drug,” while alcohol was treated as acceptable (EA1, EA6). Athletes strongly desired open conversations with team medical staff, believing that mutual lack of education undermined communication and mental‑health support. One commented that simply “being able to have an open dialogue is huge,” suggesting they would have learned more about cannabis and their own mental health if such dialogue had been possible (EA5). They also pointed to confusion within sport hierarchies, where unclear or absent cannabis policies left leagues, teams, and clinicians without consistent guidance, and argued that education should occur “at every level,” targeting trainers, team doctors, coaches, and sport psychologists who interact closely with athletes (EA7, EA8). Athletes stressed that cannabis should be understood as a plant‑based medicine. They emphasized the need for medical staff “to be educated on that narrative to shift their prior perceptions of this being a ‘bad drug’” (EA10) so practitioners can consciously consider cannabis as potential medicine, not solely a harmful substance (EA6, EA10).

### Fear of Judgment and Punishment

This theme captures fears athletes face regarding cannabis use. It focuses on stigma, punishment, and trust issues with medical staff.

#### Negative Stigma and Punishment

Stigma and punitive responses shape athletes’ experiences and behavior. Athletes feared negative judgment for using cannabis or asking questions, leading many to become secretive. One athlete linked this to stereotypes and said they refrain from telling people they use cannabis “because of fear of negative judgment” (EA10). Others worried that being caught smoking, or “even asking,” could result in being cut from the team or traded (EA5, EA6). This fear extended to medical interactions, where athletes believed staff might report them simply for showing curiosity (EA3, EA9, EA10). Concerns were reinforced by punitive experiences, such as a teammate’s dismissal for using cannabis on a day off in an environment described as “zero tolerance. . . Done, goodbye, see you,” which discouraged athletes from “investigat[ing]” cannabis because the consequences felt so severe (EA3).

Stigma influenced behavior in complex ways: most athletes hid their use, while a few described leaning into a rebellious identity, saying teenage rule‑breaking around cannabis was “tied up in that rebel aspect” and that “a lot of [the] trouble. . . came from the stigma,” which made them push against a “stupid boundary” rather than feel like worthy participants (EA5). Sources of stigma included peers, management, medical staff, family, and society, with parents warning athletes not to “ruin [their] shot” and especially judged smoking compared with tinctures or gummies, which some believed might have less stigma by reducing valid concerns about the effects of inhalation (EA5, EA9, EA10). Stigma also affected performance evaluations, with athletes worried they would be viewed as less committed if they used cannabis (EA1, EA5, EA6, EA7).

Athletes noted contexts with less stigma (such as teams or regions with legal markets) where they felt comfortable being open, especially when “the majority of [the] team used it,” compared with places where they “might not be as comfortable” disclosing use (EA1). Punitive policies and drug testing created ongoing anxiety. Athletes perceived testing and current cannabis education as reinforcing existing stigma, with lessons that only include an exaggerated stereotypical and unrealistic downward‑spiral narrative. They felt this approach was more about satisfying funders than supporting athletes, leading 1 person to complete a module as a sanction while actively using cannabis, “. . .it’s funny because I was smoking weed while doing it [the punishment educational module]. . . and then a year later, doing the same thing [smoking cannabis while watching the punishment video again]” (EA5).

Drug testing reinforced fear and raised fairness concerns, as athletes questioned why cannabis was treated like performance‑enhancing drugs and argued that if cannabis use warranted sanction, alcohol should be treated similarly given how heavy drinking was common yet less harshly judged (EA1). Overall, athletes described pervasive stigma and punishment that shaped their behavior, limited open dialogue, and discouraged even basic questions about cannabis, setting the stage for calls for more open, education‑focused approaches.

#### Complexities of Trusting Medical Staff

The subtheme “Complexities of Trusting Medical Staff” explores how athletes navigate trust in team medical staff, particularly around cannabis. Trust varied widely and was shaped by personal relationships, team hierarchy, and fears that information would be passed up to management. 

Trust in trainers developed over time and grew with how much time athletes spent with them. Athletes reported stronger relationships and better communication with staff they saw daily, especially strength and conditioning coaches, and said these friendships made it easier to discuss sensitive topics such as cannabis. One athlete noted, “It’s just their [trainers’] willingness to hang around the guys. . . it’s just how much conversations you have about anything with them” (EA2). Another elaborated, “. . . You might even end up telling them. . . I’m having trouble sleeping because I’m going through this with my family. . . it’s almost like it’s therapy” (EA8). Some athletes described highly trusting relationships with particular trainers who were open‑minded and willing to learn. These trainers were seen as counsellors, from taping ankles daily, sharing handshakes, hearing complaints about coaches, and forming “very strong” relationships that felt different from other roles in the organization (EA3, EA9). This trust depended heavily on individual trainer characteristics, however. Athletes felt safer with trainers who held positive views on cannabis or used it themselves and were wary of those seen as judgmental. One athlete explained, “It depends on the trainer. . . I wouldn’t have said anything to some because anything you say goes straight back to coach. . . The other 2 I feel like I would have felt more comfortable. . . but I would have had to preface it with ‘Please keep this between us. . . I’m just asking for a ‘friend’ type of thing’” (EA3).  Athletes relied on teammates to signal which staff could be trusted. New players reported low initial trust after “hear[ing] stories” of trainers “ratting on other matters” and were warned to “steer clear” of certain staff (EA2). Because trainers and performance staff answered to upper management, athletes believed that questions about cannabis could quickly reach senior decision‑makers and threaten their careers, leading some to say they would “definitely not” ask team medical staff about cannabis because of potential repercussions (EA6, EA2).

Concerns extended to doctors and higher‑level personnel, whom athletes saw less frequently and perceived as more likely to judge or report them. Many preferred third‑party medical providers outside the team, who had no power over contracts or playing time, and described limited trust in internal staff, saying they “wouldn’t trust [them] a whole lot with conversations regarding cannabis use” or “wouldn’t tell a soul. . . I wouldn’t trust any of them” (EA7, EA10). One athlete described opening up to an outside professional because they feared team‑affiliated staff would tell the coach, who might think there was a problem and bench them (EA3).  When asked whether players would trust internal staff more than third‑party providers, athletes consistently said they would “trust somebody outside of [the team] more for sure” and “would never go to the trainer and/or the doctor [about cannabis]” (EA7). As a result, many avoided discussing cannabis with team medical staff altogether.

Athletes perceived younger staff as more receptive and knowledgeable about contemporary issues, including cannabis. One athlete felt able to “tell [a younger coach] anything” (EA1), while another believed it would be “very hard” for older personnel to accept cannabis but expected younger staff to be more open, especially if they had grown up with different cultural views (EA6). Some athletes mentioned staff who used cannabis themselves or were clearly aware of its role in athletes’ lives and open to learning more, suggesting that as organizational attitudes shift, these younger or more progressive staff could become key partners in future cannabis education and support (EA1, EA8, EA9).

### Hiding in Plain Sight

The theme “Hiding in Plain Sight” describes the secretive nature of cannabis use among elite athletes and how medical staff also conceal their own awareness of certain pieces of sensitive information. Both groups use secrecy to protect themselves, the team, and the organization from judgment and punishment related to cannabis use.

Athletes often hid their cannabis use because of career impact concerns, avoiding conversations to “not get in trouble” (EA4, EA5). One explained the everyday tension: coming into practice “kind of looking over your shoulder” (EA2). Some athletes’ medical cannabis use was known but ignored by staff, who “just [left] it alone” rather than discuss it (EA8). Athletes believed staff avoided conversations about cannabis to protect their own jobs, choosing to “shut it down. . .because of the stigma” and fear of “get[ting] in trouble themselves” (EA3). Athletes reported medical staff were often aware of cannabis use but chose to “turn a blind eye,” believing “it was just better for both parties. . . if it [cannabis] wasn’t brought up” (EA8). Medical staff sometimes actively protected athletes from consequences by warning them before tests, saying “You better not be hitting the bong right now” (EA8, EA9). This leniency could depend on player status, producing “a 2-tiered system. . . The star athletes could do what they wanted, and non-star athletes had to follow the lines” (EA6). A “don’t ask, don’t tell” understanding was perpetuated with in many teams, where cannabis use was “a quiet thing that everybody understands” (EA1). Staff “pretty much knew” who users were while “nobody bothered us about it,” making cannabis “a lot more acceptable in a funny way, like a hush hush way” (EA5). Staff intervened only when use affected performance or created off-field problems (EA6).

### Law and Order

This final theme examines how regulatory environment shapes cannabis use and organizational responses.

#### The Influence of Rule and Law

Rules and laws guide athletes’ decisions. Many avoided cannabis during the in-season because there are rules against it. One athlete stated, “I do not use cannabis during the season because it is a banned substance” (EA10). Others focused on avoiding trouble with the league, stressing they did not want to do “anything illegal” (EA5, EA7); however, recent legalization shifted attitudes and behavior. One athlete described a legalized setting with “a thousand different places that would deliver. . .thousands of different products” (EA5). With legalization, athletes felt more comfortable experimenting and saw teammates “more willing to experiment with it. . .see the benefits” (EA2, EA1). Legal markets also reduced reliance on street dealers: “It. . . is good because you don’t have to get it [cannabis]. . . off somebody on the street” (EA2, EA1).

Despite increased openness in society, most athletes still prioritized staying within the law, like avoiding travel with cannabis because “there's still laws out there” (EA6, EA7). Geography strongly shaped comfort levels. Legal-market regions (especially the West Coast of the United States and Canada) were seen as more knowledgeable and accepting (EA9). One athlete picked the location of his sports team partly because “the weed laws. . .were really cool” and cannabis was decriminalized (EA5). In long‑legal jurisdictions, dispensary visits became “way more common” and discussed more openly, and athletes believed people would be receptive if educated “on the positive effects” (EA1, EA10). Athletes hoped ongoing legalization would foster education. Once cannabis was not viewed solely as illegal, athletes felt people were “open to a conversation” (EA8). Athletes stressed pairing any rule change with education so athletes “go about using cannabis responsibly,” seeing it as “more so about being educated” (EA10).

##### Organizational Issues

Organizational concerns about health, image, control, and performance influence cannabis management. Athletes believed organizational concerns were multifactorial. In addition to performance concerns, optics also mattered with concerns for “how it looked for the program. . .definitely, image based” (EA10, EA8, EA7). Teams feared losing control, seeing cannabis as something they “couldn't control. . .how often or how much” athletes used (EA2), and something their staff didn’t really understand. Athletes emphasized stark contrast between alcohol and cannabis. Alcohol was accepted widely and tied to team bonding: “There’s hardly any stigma. . .associated with alcohol intake” (EA10). Drinking on off days was “very normal” (EA2, EA7) despite known harms. Alcohol drew little stigma and was even praised as positive team time (EA7). Cannabis, in contrast, remained stigmatized and hard to discuss. Athletes felt much more nervous asking about cannabis than alcohol (EA3, EA4). Medical staff were perceived as afraid of getting in trouble even for talking about cannabis and were unprepared to address it. Athletes believed meaningful change must “come from the top down” (EA4). If leagues mandated education, organizations would “have people that will know this, and they would bring in the experts if they had to” (EA6).

## Discussion

This is the first qualitative study to explore elite and professional contact sport athletes’ knowledge, opinions, and perspectives regarding cannabis use. Five themes were identified: Athletes use cannabis, Education and knowledge, Fear of judgement and punishment, Hiding in plain sight, and Law and order, highlighting the complex relationship between athletes and cannabinoid use and the need for improved education, communication, and policy.

### Athletes Use Cannabis

Athletes reported using cannabis for pain management, mental health, relaxation, recovery, and sleep, consistent with literature on therapeutic benefits.^43^ Cannabis may provide an alternative to opioids for pain management,^45^ with cannabinoids reducing pain and inflammation.^3,5,27,32,33^ Studies suggest cannabis may enhance sleep quality,^6,31^ and may reduce anxiety and depression.^17,22,36,44^

Participants described deliberate, controlled use intended to avoid performance decrements, emphasizing the need to clarify performance effects and optimal dosing in athletic populations. Their approach reflects the self-discipline and regimented habits athletes typically apply to nutrition and training,^12^ but raises questions about long-term effects. Evidence suggests potential neuroprotective properties,^9,35,41^ yet chronic use, especially smoking, may be harmful.^4^ The risk-to-benefit profile of cannabinoid use in contact sport athletes and the impact of acute and chronic use on performance and athlete health remain insufficiently understood.

### Athlete Knowledge and Education

Substantial gaps in athletes’ cannabis understanding are due largely to limited formal education. Athletes typically rely on peers, the internet, and personal experimentation, which increases risk of misinformation. They view team medical staff as unreliable sources and call for education and open dialogue, consistent with evidence that few medical schools teach about cannabis use in any meaningful way,^10,16,30^ leaving both providers and athletes without adequate guidance. This deficit may foster uninformed choices and allow commercial actors to promote products with questionable claims. Athletes seek clear explanations for regulations and balanced education on risks and benefits, rather than solely stigma reinforcing messaging. Evidence-based, comprehensive programs are needed.

### Cannabis Stigma and Barriers

Fear of negative judgment was a major barrier to open discussion. Athletes described stigma, career concerns, and punitive organizational policies, revealing shortcomings in current systems and a need for confidential, nonjudgmental guidance for athletes, consistent with evidence that stigma deters people from seeking information and help.^29^ Perceived stigma can discourage disclosure, potentially worsening health outcomes if athletes avoid care. Targeted, evidence-based educational initiatives to reduce stigma are crucial for creating a supportive environment and encouraging informed decision-making.^29,43^

### Evolving Cannabis Landscape in Sports

The regulatory environment strongly shapes athletes’ attitudes and behaviors. Legalization has increased interest and acceptance, but strict in-season regulations remain a deterrent. Clear, consistent policies are needed to balance potential benefits with fair competition and athlete safety.^23^ Broader societal shifts have not eliminated discrepancies between jurisdictions and leagues,^18,24,43^ leaving athletes uncertain about sport-specific expectations.

Harmonizing policies grounded in emerging evidence will help prioritize athlete health while reflecting changing norms.^23^ Organizations must develop policies integrating health, performance, and athlete autonomy so athletes can navigate legal and regulatory complexities and make safer choices.^2^

### Organizational Issues

Organizational concerns about health, image, control, and performance heavily influence cannabis management in sport. The contrasting acceptance of alcohol versus cannabis underscores the need for a more balanced approach that weighs relative risks and benefits of both substances.^19^ Normalizing alcohol while stigmatizing cannabis creates confusion and erodes trust, even as informal conversations suggest movement toward more open dialogue.^26^

The reported lack of trust and open communication between athletes and medical staff is particularly concerning. Strong patient-provider communication is critical in healthcare, including around cannabinoid products.^1,40^ Improving communication channels so athletes feel safe raising cannabis questions enhances care quality and ensures accurate information. Nonjudgmental spaces for discussion are vital for effective care and for aligning organizational practice with current evidence and athlete needs.^13,34^

## Implications for Practice and Policy

This study suggests several important directions: first, cannabis education tailored to athletes and sports medicine.^20,25,39^ Second, policies should be harmonized across leagues and grounded in emerging evidence, with clear communication. Third, organizations should foster open, nonjudgmental communication between athletes and medical staff.^11^ Fourth, leagues should reduce stigma surrounding cannabis use in sport. Finally, professional leagues should support further research on cannabis effects on athletic wellness, performance, and long-term health outcomes.

## Limitations and Future Directions

This study offers insight into professional athletes’ perspectives on cannabis but has limitations. The small sample (n = 10) may not represent all athletes, although it provides an initial view of elite contact sport athletes’ experiences. The focus on elite contact sports may also limit generalizability to other athletic contexts; future research should include a broader range of sports and larger, more diverse samples, including noncontact athletes. Self-selection bias is a limitation, as participants volunteered and may have been more willing to discuss cannabis with a positive viewpoint. Future work should incorporate quantitative studies assessing prevalence, longitudinal research on long-term effects on performance, and intervention studies evaluating educational programs.

## Conclusion

This study highlights the complex relationship between professional athletes and cannabis use in professional and college-level contact sports. It shows growing interest in cannabinoid products and the importance of addressing this safely and appropriately in athletics. The substantial stigma and lack of knowledge among medical personnel working with these athletes underscores the need for improved education, communication, and evidence-based policies to protect athlete health in an evolving legal and commercial cannabis landscape.

## Supplemental Material

sj-docx-1-sph-10.1177_19417381261460194 – Supplemental material for Inside the Minds of Professional and Elite Athletes: A Qualitative Exploration of Perspectives on Cannabinoid Use in Contact Sports Supplemental material, sj-docx-1-sph-10.1177_19417381261460194 for Inside the Minds of Professional and Elite Athletes: A Qualitative Exploration of Perspectives on Cannabinoid Use in Contact Sports by Elizabeth S. Thompson, Jane Alcorn, Robert B. Laprairie, Noelle Rohatinsky, Payam Dehghani and J. Patrick Neary in Sports Health
