# Supplementary material for: Inside the Minds of Professional and Elite Athletes: A Qualitative Exploration of Perspectives on Cannabinoid Use in Contact Sports
Source: Sports Health. 2026 Jul 7:19417381261460194. Online ahead of print. doi: 10.1177/19417381261460194 (PMC13346102; doi:10.1177/19417381261460194)
Supplement: sj-docx-1-sph-10.1177_19417381261460194 – Supplemental material for Inside the Minds of Professional and Elite Athletes: A Qualitative Exploration of Perspectives on Cannabinoid Use in Contact Sports [file sj-docx-1-sph-10.1177_19417381261460194.docx]

**Supplement 1: Interview Question Guide**

**Exploring the knowledge, opinions, and perspectives of elite contact sport athletes and medical staff regarding *Cannabis* use:**

**Interview Questions**

**Athlete Interview Questions**

1. Tell me about your personal experience using cannabis in and out of season.

2. Tell me about any stigma you may have felt regarding use or inquiry into use.

3. What do you think is the greatest concern from upper management regarding cannabis use? (i.e., your health or liability, perception and disciplinary actions?)

4. If used, what are/were you specifically using it for (i.e. therapeutic or recreational purposes included)? How did you use it? Was your experience positive or negative? (i.e. Do you feel it helps/helped with this issue?)

5. Why did you want to explore cannabis as a substance? (i.e. Were you initially using a different substance to address this issue before trying cannabis?)

6. Where did you find information to choose your cannabis product, the administration route, and the dose? (If no, have you ever looked for info on cannabis use? Where did you look?)

7. Tell me about what response you think you might receive from medical/training staff if you were to inquire about cannabis use OR Tell me about the medical staff or trainers response. (negative, positive, or neutral response?) to your inquiry.

8. Do you think they would have the same response to alcohol question/convo? Why?

9. What type of ‘information’ did you discuss with the medical staff or trainers before using cannabis. What information did they offer?

10. Tell me about your confidence level confiding in medical staff regarding cannabis use (also, do you (did you) feel comfortable confiding in them about this topic?)

11. How qualified and educated do you feel your medical staff and trainers are to provide cannabis care advice?

12. If we were educating team staff members, who do you feel would be most appropriate staff member to teach?

13. Did you play before or after your State (USA) or Country (e.g., Canada) legalized cannabis? Tell me how this influenced your approach to using cannabis?

14. Is there anything you would like to talk about that I have not asked or addressed?
